# Linking Innate and Adaptive Immunity: Human V*γ*9V*δ*2 T Cells Enhance CD40 Expression and HMGB-1 Secretion

**DOI:** 10.1155/2009/819408

**Published:** 2009-10-13

**Authors:** Shirin Kalyan, Anthony W. Chow

**Affiliations:** Division of Infectious Diseases, Departments of Medicine, Microbiology and Immunology, University of British Columbia and Vancouver Hospital Coastal Health Research Institute, Vancouver, BC, Canada V5Z 3J5

## Abstract

*γδ* T cells play an important role in regulating the immune response to stress stimuli; however, the mean by which these innate lymphocytes fulfill this function remains poorly defined. The main subset of human peripheral blood *γδ* T cells responds to nonpeptidic antigens, such as isopentylpyrophosphate (IPP), a metabolite in the mevalonate pathway for both eukaryote and prokaryote cells. IPP-primed *γδ* T cells significantly augment the inflammatory response mediated by monocytes and *αβ* T cells to TSST-1, the staphylococcal superantigen that is the major causative agent of toxic shock syndrome. Here we show that the small pool of activated peripheral *γδ* T cells induces an early upregulation of CD40 on monocytes and the local release of High Mobility Group Box-1 (HMGB-1), the molecule designated as the late mediator of systemic inflammation. This finding provides a new basis for how *γδ* T cells may serve as influential modulators of both endogenous and exogenous stress stimuli.

## 1. Introduction

*γ* *δ* T cells have a nonredundant role in regulating immune homeostasis. In comparison to *αβ* T cells, they lack receptor diversity yet demonstrate remarkable plasticity in response to both infectious and noninfectious stimuli. As an extension of their dichotomous nature, *γδ* T cells have been held liable for both instigating over-zealous immune responses [1–3] as well as reigning in inflammation by controlling activated macrophages and promoting wound healing [4–6]. In humans, the main subset of peripheral blood *γδ* T cells usually comprises between 1 and 5% of circulating T cells and bears the canonical V*γ*9V*δ*2 T cell receptor. These V*γ*9V*δ*2 T cells are unique to primates, and they respond to endogenous and exogenous stress-induced molecules that are nonpeptidic in nature and preclude the requirement for processing or presentation by classical MHC molecules [7–9]. Isopentylpyrophosphate (IPP) is the prototypical phosphoantigen recognized by V*γ*9V*δ*2 T cells that was first isolated from lysates of mycobacteria [7]. It was later determined to be a metabolite in the mevalonate pathway in all eukaryotic and some prokaryotic cells, including *Staphylococcus aureus* [10, 11]. During the initial phase of infection with low bacterial inocula of *S. aureus*, V*γ*9V*δ*2 T cells are activated by endogenous mevalonate metabolites through the accumulation and dephosphorylation of the hydroxymethylglutaryl-coenzyme A reductase, the rate-limiting enzyme of the mevalonate pathway [11]. Therefore, primates have evolved the ability to readily respond to bacterial infection as well as endogenous damage by sensing the dysregulation of the mevalonate pathway, which may be considered a stress-associated “alarmin” for these primordial lymphocytes.

We have shown that stimulating the small pool of human *γδ* T cells present in PBMC with IPP markedly augmented the early proinflammatory response to Toxic Shock Syndrome Toxin-1 (TSST-1) [3]. TSST-1 is a staphylococcal superantigen that exerts a potent inflammatory response by bridging V*β*2 specific *αβ* T cell receptors with conserved regions of the MHC class II molecules of antigen presenting cells (APCs) outside the peptide-binding groove [12]. Ferry et al. [13] demonstrated the presence of specific V*β* T cell signatures of various superantigenic toxins, including TSST-1, in the blood of patients with staphylococcal TSS. We previously demonstrated that IPP-activated *γδ* T cells augment the levels of proinflammatory cytokines within the first 6 hours post-TSST-1 treatment, thereby changing the kinetics and magnitude of the immune response to the superantigen [3]. We have also shown that High Mobility Group Box Protein-1 (HMGB-1) is released following TSST-1 stimulation [14]. HMGB-1 is a highly conserved nonhistone DNA-binding protein found in virtually all nucleated cells and is regarded as being the late mediator of sepsis [15]. Activated monocytes secrete HMGB-1 after exposure to endotoxin [16], and it is detectable in the serum of septic patients within 24 hours. Importantly, neutralizing the effects of HMGB-1 at this late time point, either by anti-HMGB-1 antibodies or antagonists, rescued mice from sepsis-induced mortality [15]. In contrast to endotoxin, which activates only macrophages to secrete HMGB-1, both T cells and macrophages participate in the nuclear translocation and subsequent release of HMGB-1 into the extracellular environment following TSST-1 stimulation [14]. 

The first aim of the present study was to evaluate the influence exerted by IPP-primed V*γ*9V*δ*2 T cells on the change in costimulatory molecule expression that could explain their ability to enhance the immune response to TSST-1. The second objective was to determine the consequence of *γδ* T cell mediated immune modulation on the expression and secretion of HMGB-1.

## 2. Materials and Methods

### 2.1. Toxin Purification

Recombinant TSST-1 was purified from culture supernatants of *S. aureus *strain RN4220 previously transformed to carry the *tst* gene, using both preparative isoelectric focusing and chromatofocusing [17]. Toxin purity was assessed by silver staining after sodium dodecyl sulfate-polyacrylamide gel electrophoresis on 14% acrylamide gels, and LPS activity was undetectable by the *Limulus *amoebocyte lysate gelation (sensitivity limit, 10 pg/mL).

### 2.2. Preparation of Cells and Culture Conditions

**Figure 1:**
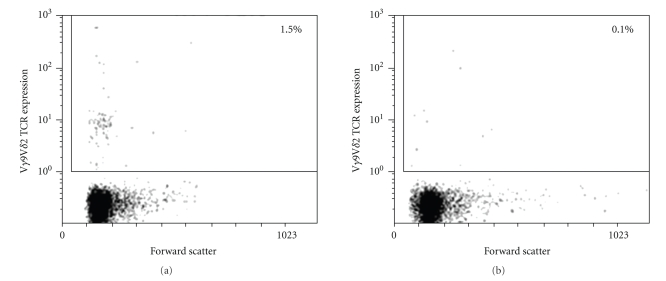
Flow cytometry plot showing a representative population of *γδ* T cells present in human PBMCs. Before depletion by magnetic bead separation, *γδ* T cells constituted ~1.5% of PBMC (a) and <0.1% after depletion (b).

Fresh human peripheral blood mononuclear cells (PBMC) from healthy donors were obtained by Ficoll-Paque PLUS (Amersham Biosciences Corp., Piscataway, NJ) density centrifugation, and cultured in 96-well U-bottom plates at 1.5 × 10^6^ cells/ml in complete culture medium consisting of RPMI 1640 (StemCell Technologies Inc., Vancouver, BC, Canada), 10% heat-inactivated fetal bovine serum (HyClone Laboratories Inc., Logan, UT), 2 mM L-glutamine (StemCell), 25 mM Hepes buffer (StemCell), and 2 ug/ml of polymyxin B sulphate (Sigma-Aldrich Corp., St. Louis, MO). Depletion of *γδ* T cells was accomplished by immunomagnetic positive cell selection with an anti-*γδ* TCR MAb (clone Immu510, BD BioSciences Pharmingen Inc., San Diego, CA) conjugated to an antidextran MAb tetramer (StemCell), which left the rest of the PBMC untouched, using the StemSep protocol (StemCell). Purity of the *γδ* T cell-depleted PBMC was analyzed by flow cytometry using phycoerythrin-(PE-) conjugated anti-V*δ*2 antibody (BD BioSciences Pharmingen). Figure 1 shows the population of V*γ*9V*δ*2 T cells in PBMCs before (which made up 1.5% of live cells) and after depletion (0.1%).

### 2.3. Treatment of PBMC with IPP and TSST-1

PBMCs with or without *γδ* T cell depletion were treated with either 45 *μ*M IPP (Sigma-Aldrich) or left untreated in complete growth medium for 16–18 hours (overnight) at 37°C, 5% CO_2_ prior to treatment with 1 nM TSST-1. For secreted HMGB-1 detection, culture supernatants were microcentrifuged at 800 × g for 5 minutes and frozen at −70°C until analysis.

### 2.4. Flow Cytometric Analysis of Surface-Expressed HMGB-1 on Differentiated Cells and Expression of Costimulatory Molecules

Surface expressed HMGB-1 was analyzed by flow cytometry (FACSCalibur Flow Cytometry System, BD BioSciences Pharmingen) using the anti-HMGB-1 antibody and secondary Alexa488-conjugated antibody (as described below). Costimulatory molecule cell surface expression was performed by incubating cells at 4°C (protected from light) for 30 minutes with the following fluorescently-conjugated antibodies: APC antihuman HLA-DR (LN3), PE-Cy5 anti-human CD86 (B7-2) (eBioscience, Inc), FITC anti-CD40 (5C3), PE-Cy5 anti-CD80 (B7-1), PE-Cy5 anti-CD28 (CD28.2), and APC anti-CD25 (M-A251) (BD Biosciences, PharMingen). Cells were subsequently washed, resuspended in buffer containing 2 mM EDTA (to facilitate detachment of adherent cells), and transferred to FACS to analyze by flow cytometry (a minimum of 10 000 events were collected).

### 2.5. Fluorescent Microscopy Analysis

Surface expression of HMGB-1 on PBMC by fluorescent microscopy has been previously described in detail [14]. In brief, PBMCs were cultured (37°C, 5% CO_2_) directly on sterile cover slips to allow attachment of adherent cells overnight before stimulation with 1 nM of TSST-1. For HMGB-1 expression, cells were incubated with rabbit anti-human HMGB-1 IgG as previously described [14], diluted in blocking buffer (PBS, 3% FBS), followed by a secondary Alexa488-conjugated goat antirabbit IgG antibody. Cells were subsequently fixed using Cytofix buffer (Pharmingen). Fluorescently labelled PBMCs were then stained with Hoechst 3342 nuclear dye (Molecular Probes) according to manufacturer's directions, and mounted on slides using Prolong Anti-Fade reagent (Molecular Probes). Cells were visualized with an AxioPlan II fluorescence microscope equipped with a CCD camera using Northern Eclipse software (Epix) for acquisition of images. Images were taken with the 63 x oil immersion objective lens, and Adobe Photoshop 6.0 software was used for image layout.

### 2.6. Western Blot Analysis of Secreted HMGB-1

PBMC culture supernatants were concentrated (~10-fold) from the original volume of 1.5 mL using Amicon Ultra centrifugal filters with a molecular weight cutoff of 10 kDa (Millipore). The supernatants then had been further prepared using the SDS-PAGE Clean-up kit (Amersham) according to the manufacturer's directions prior to running on a 12% polyacrylamide gel. Western blotting was performed by semidry transfer of proteins (Trans-Blot SD Semi-Dry Electrophoretic Transfer Cell, BioRad) onto an Immobilon-P PVDF membrane (Millipore) which was blocked for 1 hour at room temperature with 1% BSA, 0.5% Tween in TBS prior to overnight incubation (at 4°C) with rabbit polyclonal anti-HMGB-1 antibody. The membrane was subsequently incubated with anti-rabbit IgG horse-radish-peroxidase-(HRP-) conjugated secondary antibody for 1 hour at room temperature on a shaker. HMGB-1 detection was performed using Super Signal substrate (Pierce) and developed as well as analyzed using the Alpha Innotech 3400 Gel Documentation system (Alpha Innotech).

### 2.7. Statistical Analysis

Statistical analysis was performed using Prism 4.0 software package (GraphPad Software, Inc., San Diego, CA). Student *t*-tests (one-tail) were used to assess the significance of differences in HMGB-1 and co-stimulatory molecule expression between IPP treated and untreated PBMC at different times post-TSST-1 stimulation. The effects of IPP over time on co-stimulatory molecule expression by APC (CD40, CD80, CD86, HLA-DR) and *αβ* T cells (CD25, CD28) were further examined by 2-way ANOVA. Differences were considered significant if the probability of the null hypothesis was less than five percent (*P* < .05). Each experiment represents 3 to 4 healthy subjects unless otherwise noted.

## 3. Results

### 3.1. IPP-Primed *γδ* T Cells Induce an Early Upregulation of CD40 following TSST-1 Stimulation

**Figure 2:**
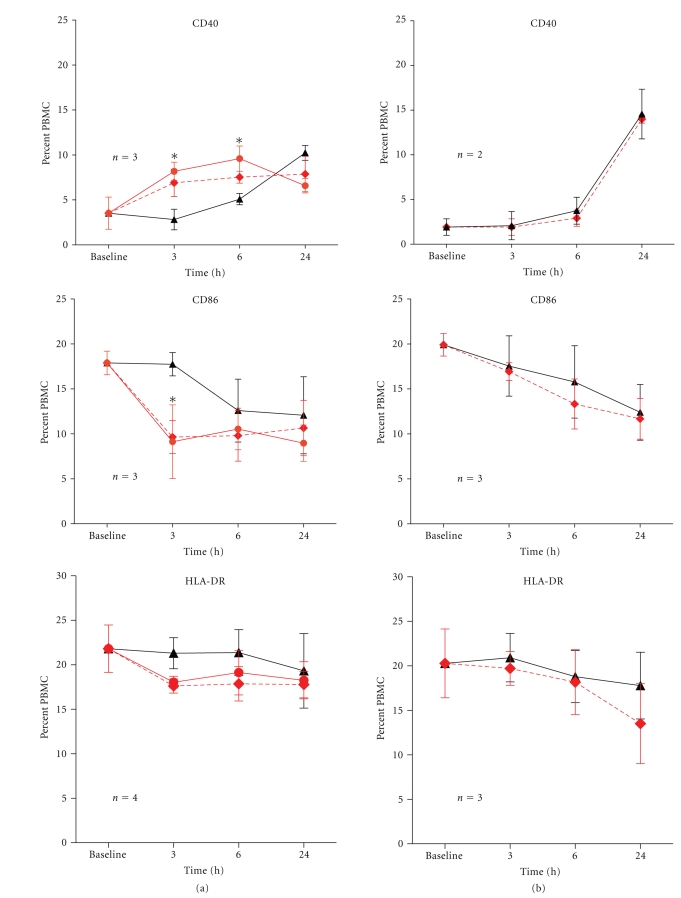

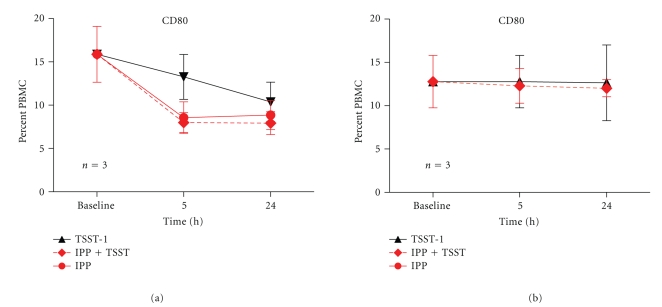
Modulation of CD40, CD86, HLA-DR and CD80 on APCs by IPP-activated *γδ* T cells. PBMC were cultured overnight in the presence of IPP or absence of IPP before stimulation with 1 nM of TSST-1 (a). Data (mean ± SEM) for expression of CD40, CD86, HLA-DR and CD80 were shown after various time intervals following TSST-1 stimulation, and the numbers of healthy donors studied for each co-receptor are as indicated. Baseline values were obtained from PBMC treated with RPMI growth medium alone. To determine the influence of IPP-activated *γδ* T cells on receptor expression following TSST-1 stimulation, comparisons were made between IPP + TSST-1 (dashed red lines) versus TSST-1 alone (solid black line), and significant differences were denoted by (∗). To confirm that any observed difference in receptor expression was due to the presence of activated *γδ* T cells, a cognate group of PBMC were depleted of *γδ* T cells (<0.1%) and studied in parallel experiments (b). To determine the influence of TSST-1 on IPP-activated *γδ* T cells, comparisons were made between IPP + TSST-1 versus IPP alone. Overnight IPP treatment significantly up-regulated CD40 expression at 3hours and 6hours after TSST-1 stimulation (*P < .05, student's *t* test), and down-regulated CD86 expression at 3h after TSST-1 stimulation (*P < .05). No other significant differences in receptor expression were observed for the other co-stimulatory molecules.

Following overnight treatment with IPP, CD40 expression was upregulated in comparison to those that were untreated (IPP + TSST-1 versus TSST-1 alone; *P* < .05, Student's *t*-test) (Figure 2(a)). The elevated CD40 expression was observed up to 6 hours postsuperantigen stimulation, after which time CD40 was downregulated or internalized. We previously demonstrated that CD40L (CD154) is expressed on T cells within 3 hours following exposure to TSST-1 [18], therefore, the down-regulation of CD40 beyond 6 hours is likely a consequence of CD40 binding to CD40L on TSST-1-activated T cells. We verified that up-regulation of CD40 expression in populations exposed to IPP was solely attributed to the presence of activated *γδ* T cells, since removal of *γδ* T cells completely abrogated the effect of IPP (Figure 2(b)). The effect of IPP-activated *γδ* T cells over time on CD40 expression following TSST-1 stimulation was further verified using 2-way ANOVA (IPP x time interaction, *P* < .05). In contrast to CD40, the expression of CD86 was down-regulated early in IPP treated PBMC at 3 hours following TSST-1 stimulation (IPP + TSST-1 versus TSST-1 alone; *P* < 0.05, Student's *t*-test) (Figure 2(a)). Again, this effect was abrogated following depletion of *γδ* T cells (Figure 2(b)). Two-way ANOVA confirmed an interaction effect (IPP x time interaction, *P* < .05). There was a similar trend for decreased expression of HLA-DR (the MHC class II molecule to which TSST-1 binds on APC) and CD80 by IPP treatment, but the differences were not statistically significant.

### 3.2. TSST-1, but Not IPP-Primed *γδ* T Cells, Activate *αβ* T Cells

**Figure 3:**
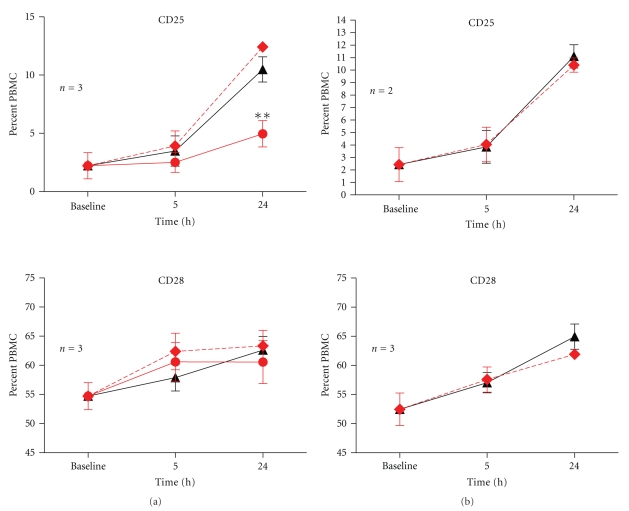
Modulation of CD25 and CD28 on *αβ* T cells by IPP-activated *γδ* T cells. PBMCs were cultured overnight in the presence of IPP or absence of IPP before stimulation with 1 nM of TSST-1 (a). Data (mean ± SEM) for expression of CD25 and CD28 were shown after various time intervals following TSST-1 stimulation, and the numbers of healthy donors studied for each coreceptor were as indicated. Baseline values were obtained from PBMC treated with RPMI growth medium alone. To determine the influence of IPP-activated *γδ* T cells on receptor expression, comparisons were made between IPP + TSST-1 (dashed red lines) versus TSST-1 alone (solid black line) and significant differences were denoted by (∗). To confirm that any observed difference in receptor expression was due to the presence of activated *γδ* T cells, a cognate group of PBMCs was depleted of *γδ* T cells (<0.1%) and studied in parallel experiments (b). To determine the influence of TSST-1 on IPP-activated *γδ* T cells, comparisons were made between IPP + TSST-1 versus IPP alone, and significant differences were denoted by (∗ ∗). There was no additional effect of IPP-activated *γδ* T cells on CD25 and CD28 expression following TSST-1 stimulation; however, CD25 expression on *αβ* T cells following TSST-1 stimulation in the presence of IPP-activated *γδ* T cell was significantly higher at 24 hour than with IPP alone (**P = .001, student's *t*-test).

In contrast to the direct affect of IPP on monocyte function, there was little notable direct influence on the activation state of *αβ* T cells as evaluated by the expression of CD25 or CD28, which remained largely unchanged in response to IPP pretreatment (Figure 3). The upregulation of CD25 on *αβ* T cells at 24 hours was solely dependent on TSST-1 stimulation regardless of IPP, as shown by the comparison of IPP + TSST-1 versus IPP alone (*P* = .001, Student's *t*-test, Figure 3). Two-way ANOVA confirmed this effect (TSST-1 effect, *P* < .01; TSST-1 x time interaction, *P* < .005).

### 3.3. IPP-Primed *γδ* T Cell Potentiates the Release of Cell Surface Expression of HMGB-1 (Amphoterin) into the Extracellular Milieu following TSST-1 Stimulation

**Figure 4:**
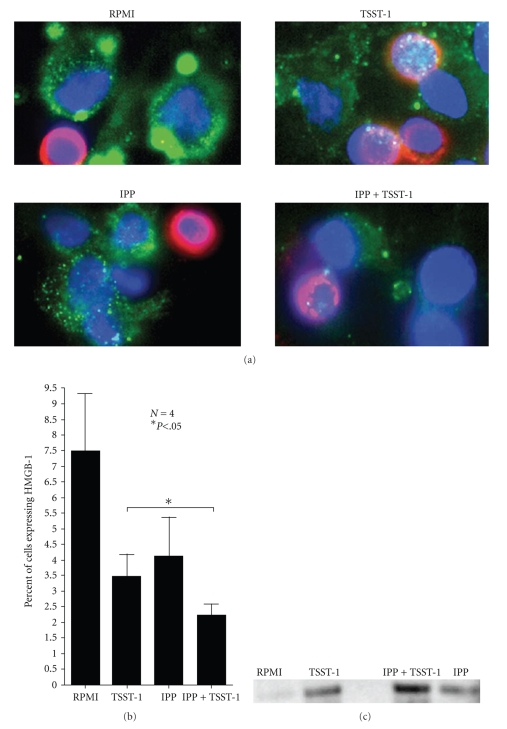
Change in cell surface expressed HMGB-1 (amphoterin) on adherent monocytes in PBMC following IPP and TSST-1 treatment. (a) Fluorescent microscopy of surface-expressed HMGB-1 in PBMCs. HMGB-1 is shown in green, T cells in red, and nucleus in blue. Top left panel—Resting PBMC cultured on cover slips for 48 hours were stained for surface expressed HMGB-1. Top right panel—Surface expression of HMGB-1 after 24 hour culturing in growth medium followed by 24 hour TSST-1 treatment. Bottom left panel—Surface expression of HMGB-1 48 hours after IPP treatment. (Bottom right panel) Surface expression of PBMC that had been treated overnight with IPP and subsequently treated with TSST-1 for 24 hour. (b) Bar graph of the flow cytometry analysis quantifying the release of HMGB-1 from the cell surface (*N* = 4; error bars represent SD). Statistic shown compares IPP + TSST-1 versus TSST-1 alone (*P* < .05, paired Student's *t*-test). (c) Western blot analysis of secreted HMGB-1 in cultured supernatants.

Rouhiainen et al. established that monocytes that had become adherent export HMGB-1 to the cell surface where it serves a function for process extension, migration, and cell-cell interaction [19]. Extracellular cell-surface expressed HMGB-1 has been called amphoterin to differentiate it from intracellular HMGB-1 found in the nucleus [19]. Given the direct influence of *γδ* T cells on the activation, maturation, and regulation of cells of monocytic lineage [20, 21], we wanted to determine how IPP stimulation would influence the surface manifestation of HMGB-1 on cultured monocytes present in PBMC. Figure 4(a) shows that resting adherent cells have HMGB-1 (stained in green) localized at focal points around the perimeter in vesicle-like pockets (top left corner; RPMI). T cells, which are nonadherent cells in suspension (stained in red), do not express HMGB-1 on the surface in this manner. Adherent monocytes bearing surface expressed HMGB-1 subsequently released it following TSST-1 stimulation (Figure 4(a); top right panel). Overnight treatment with IPP alone also led to a change in extracellular HMGB-1 expression in PBMC cultures (Figure 4(a); bottom left panel). There was an observable modification on both the morphological appearance of macrophages as well as the distribution of HMGB-1 around the perimeter, which became less concentrated in vesicle-like foci and more dispersed. Overnight incubation of PBMCs with IPP followed by TSST-1 stimulation resulted in significantly more HMGB-1 being released into the extracellular environment compared to TSST-1 alone (Figure 4(a), upper and lower right panel). This change in surface expressed HMGB-1 was assessed by flow cytometry, which enabled us to both confirm and quantify the release of HMGB-1 from differentiated monocytes following TSST-1 stimulation. Figure 4(b) displays the difference between the various treatment conditions on the loss of cell-surface expressed HMGB-1 assessed by flow cytometry. IPP-pretreated PBMCs that were subsequently stimulated with TSST-1 released more HMGB-1 from the cell surface (i.e., less HMGB-1 expression on the cell surface) than those cells that were left untreated with IPP prior to superantigen stimulation (IPP + TSST-1 versus TSST-1 alone, *P* < .05, paired Student's *t*-test). The actual secretion of HMGB-1 into the extracellular milieu from TSST-1 stimulated PBMC was further verified by Western blot of cell culture supernatants (Figure 4(c)), which again substantiated that IPP pretreatment followed by TSST-1 stimulation led to its greater release than PBMC stimulated with TSST-1 without IPP-activation of *γδ* T cells (IPP + TSST-1 versus TSST-1 alone, *P* < .05, paired Student's *t*-test).

## 4. Discussion

Overnight treatment with IPP resulted in a notable up-regulation of CD40 on monocytes in the presence of *γδ* T cells. We previously demonstrated that impeding the CD40-CD40L interaction with CD40 monoclonal antibodies (but not the CD86 or CD80 interaction) completely blocks TSST-1-induced inflammation [18]. The early up-regulation of CD40 on monocytes by IPP-primed *γδ* T cells in the current study provides a basis for the prior observation that TNF*α* and IFN*γ* levels increased 3-to 4-fold during the early phase of the immune response to TSST-1 in PBMC containing activated *γδ* T cells [3]. This early augmentation was also abrogated after depletion of *γδ* T cells [3]. This study also demonstrates that the exaggerated response to TSST-1 by activated *γδ* T cells is through the direct modulation of the responsiveness of monocytes and not *αβ* T cells since we found that the upregulation of CD25 on *αβ* T cells was affected only by stimulation with TSST-1 and not IPP. The regulation of CD40 expression by activated *γδ* T cells appears to parallel the mechanism of linking innate to adaptive immunity by glycolipid responsive V*α*14 + NK T cells stimulated with *α*-galactosylceramide. It was found that the rapid release of TNF*α* and IFN*γ* to ovalalbumin, acquisition of CD4+ and CD8+ T cell immunity, and maturation of dendritic cells were all dependent on the CD40-CD40L interaction induced by the priming of V*α*14+ NK T cells by *α*-galactosylceramide [22]. In contrast to CD40, CD86 expression was found to be reduced in PBMC treated with IPP overnight. This monocytic phenotype may be reflective of a state described as “immature dendritic cells” in which cycling of the HLA-DR molecule is rapid and levels of CD86 are relatively low [23]. Taken together, these studies suggest that innate T lymphocytes responsive to specific “alarmins” influence the ability of APC to effectively stimulate cells of adaptive immunity, and the CD40-CD40L interaction likely plays a pivotal role in this process. This scenario is in agreement with recent demonstrations that *γδ* T cells induce early dendritic cell maturation and share an intimate relationship with APC [21, 24, 25]. Eberl et al. [26] further provided evidence that human *γδ* T cells are activated by microbial mevalonate metabolites and interact with monocytes and local macrophages to drive the acute inflammation in bacterial infections. Thus, *γδ* T cells provide a direct link between innate and adaptive immunity. It should be noted, however, that these studies primarily used either expanded *γδ* T cell lines or a large number of purified *γδ* T cells in coculture conditions. The conditions used in this study may be more reflective of the physiological state in which a small number of *γδ* T cells present in peripheral blood would be able to immediately respond to their stress-associated antigens, such as IPP. This early event would preclude the possibility of any expansion of *γδ* T cells, but it would lead to the efficient priming of APC.

This study also provides the first demonstration that IPP-primed human peripheral blood *γδ* T cells influence the secretion of HMGB-1 by macrophages. This finding is similar to the work by Semino et al. [27] who demonstrated that NK cells reciprocally activate autologous dendritic cells, and this interaction was also mediated through the secretion of HMGB-1. It is feasible that the enhanced HMGB-1 secretion observed in cells containing activated *γδ* T cells could be in response to an increase in apoptosis [28] due to the fact that IPP-stimulated *γδ* T cells are particularly susceptible to activation-induced cell death [29, 30] as a self-limiting precaution. During apoptosis, HMGB-1 is normally tightly bound to nucleosomes [28] as opposed to necrosis where HMGB-1 is released from cells [31]. However, work by Urbonaviciute et al. [28] showed that biologically active HMGB-1 may also be released from apoptotic cells. One of the main ligands for extracellular HMGB-1 is the receptor for advanced glycation end (RAGE) products, a member of the Ig superfamily. The expression of RAGE increases following activation on both dendritic cells and antigen stimulated T cells [32]. The release of HMGB-1 and its subsequent binding to RAGE is required for the clonal expansion, survival, and functional polarization of naive T cells [33]. 

Both dendritic cell maturation and Th1 polarization are events that have been attributed to the activity of *γδ* T cells and HMGB-1, respectively [21, 34–36]. Our findings connect these two previously unrelated concepts and brings forth the suggestion that *γδ* T cells mediate this effect and link innate and adaptive immunity by influencing the early upregulation of CD40 expression and release of HMGB-1 from APC, thereby priming the immune response to subsequent stimuli.
